# Heat and socioeconomic deprivation compound to drive coronary heart disease in Los Angeles

**DOI:** 10.3389/fpubh.2026.1784078

**Published:** 2026-03-09

**Authors:** Shutong Huo, Tessa R. Pulido, Reginald S. Archer, Joshua B. Fisher, Jason A. Douglas

**Affiliations:** 1 Department of Health, Society, and Behavior, Joe C. Wen School of Population and Public Health, University of California, Irvine, Irvine, CA, United States; 2 Department of Environmental Sciences, Tennessee State University, Nashville, TN, United States; 3 Schmid College of Science and Technology Chapman University, Orange, CA, United States

**Keywords:** coronary heart disease, health equity, land surface temperature, socioeconomic deprivation, spatial analysis, urban heat

## Abstract

**Background:**

Socioeconomic deprivation and environmental heat exposure each increase cardiovascular risk, yet evidence is limited on how these stressors co-occur and jointly shape disease burden within cities. Mapping their overlap can inform equity-oriented planning and needs-based allocation of health and social protection resources.

**Methods:**

We conducted an ecological geospatial analysis of 2,513 census tracts in Los Angeles County. Adult coronary heart disease (CHD) prevalence was obtained from CDC Population Level Analysis and Community Estimates (2021). Socioeconomic deprivation was measured using the Social Deprivation Index (SDI), and heatwave surface heat hazard was measured using land surface temperature (LST) retrieved from the ECOsystem Spaceborne Thermal Radiometer Experiment on Space Station. We identified hot spots and overlaps using Getis–Ord Gi^*^ statistics. Associations between SDI, LST, and CHD were estimated using z-score–standardized OLS regression with tract-level sociodemographic controls; we assessed spatial dependence and applied geographically weighted regression (GWR) with adaptive bandwidths to characterize spatial heterogeneity.

**Results:**

CHD hot spots overlapped with high-deprivation and high-heat areas, concentrated in the south and east of Los Angeles County. In standardized OLS models (z-scored outcome and predictors), a 1-SD increase in SDI was associated with a 0.163-SD higher CHD prevalence (*p* < 0.001), and a 1-SD increase in LST was associated with a 0.070-SD higher CHD prevalence (*p* < 0.001). GWR revealed substantial geographic variation: the SDI–CHD association was strongest in central and southern tracts, whereas the LST–CHD association was strongest in central and eastern tracts, suggesting that dominant risk drivers and intervention priorities differ by neighborhood.

**Conclusion:**

To address this, socioeconomic deprivation and urban heat indicators should be used to inform transparent resource allocation for chronic disease prevention and management. Neighborhoods with high deprivation and heat exposure should be prioritized for cooling infrastructure, home heat mitigation, and urban greening initiatives. Finally, public health, housing, and social services must work together to effectively address the structural drivers of cardiovascular disease disparities.

## Introduction

Health disparities, defined as the health differences that adversely affect low-income communities and communities of color (e.g., Black, Latiné), are a persistent and pervasive global issue (1). In the United States, racial and ethnic health disparities significantly impact community well being and quality of life (2, 3). Deeply rooted in the framework of structural racism—a system characterized by public policies, institutional practices, cultural representations, and other norms that collectively perpetuate racial inequities—these disparities manifest in stark differences in access to health-promoting resources and various health outcomes (4–7). For example, historical redlining and related housing policies restricted credit and public investment in marginalized neighborhoods, shaping today's patterns of deprivation, housing quality, and environmental exposures that contribute to health risk (8, 9). Many residents of communities of color live in historic redlining areas and are burdened by unequal access to healthcare and recreational facilities, along with increased exposure to environmental hazards (8, 10–12). These negative environmental factors further elevate the risk of cardiovascular diseases and a range of other illnesses within underserved communities of color (13–15).

Social deprivation is deeply entrenched in communities of color due to structural racism, which leads to geographic concentration of poverty and reinforces socioeconomic disparities (16, 17). Social deprivation refers to the lack of essential resources, opportunities, and social connections (18). These deficits shape daily living conditions and limit access to health-promoting environments and services (19, 20). Thus, social deprivation is strongly associated with poor health outcomes, including both physical and mental health, beyond the effects of income poverty alone (20–24). Additionally, communities of color often experience unequal access to healthcare services, including specialty care provided by healthcare professionals with advanced training and expertise in specific areas, which contributes to greater utilization of emergency services and worse health outcomes (20, 22). Geographical variations in social deprivation contribute to differences in cardiovascular disease prevalence and outcomes, with areas of higher deprivation showing increased cardiovascular disease rates (25). Higher levels of social deprivation are associated with less favorable profiles of cardiovascular disease risk factors, such as higher rates of smoking, obesity, and physical inactivity (26, 27). This framing shifts the focus from individual risk to modifiable levers that sit at the intersection of public health and social policy.

Environmental conditions also play a significant role in shaping health outcomes and disparities (28, 29). Among these, heat is a critical factor that intersects with socioeconomic conditions to further influence the health of populations living in deprived areas (30, 31). Neighborhoods historically subjected to social deprivation and low socioeconomic status experience higher heat exposure (31, 32). Individuals residing in hotter, less vegetated urban areas face a greater risk of morbidity and mortality due to elevated ambient temperatures (30, 33, 34). Especially, older adults, children, and individuals with pre-existing conditions are more vulnerable to elevated temperatures (34). In addition, heat vulnerability in the US is higher in historically redlined and contemporary disadvantaged census tracts and communities of color, with non-Hispanic African American and Black race/ethnicity groups being most vulnerable (35). Regarding cardiovascular disease, a study found that a 1 °C increase in temperature correlates with a 3.4% rise in cardiovascular mortality (36). High temperatures may be linked to increased cardiovascular disease-related emergency department visits and hospitalizations. (37, 38) However, limited research connects social deprivation with heat and racial health disparities, particularly concerning cardiovascular diseases at the ecological level.

To address this literature gap, this geospatial study examines the distribution of and associations between social deprivation, heat, and cardiovascular disease in Los Angeles County, the most populous county in the U. S. As such, Los Angeles provides an ideal setting for examining the spatial relationship between social deprivation, heat, and health outcomes, given its diverse socioeconomic landscape, significant environmental challenges, and pronounced racial and ethnic health disparities (39, 40). Additionally, coronary heart disease (CHD) is the leading cause of death in Los Angeles County, and it was highest among Black males (40). Thus, findings from this study will have important implications for Los Angeles and other urban areas facing similar challenges. By producing tract-level evidence on where risks concentrate and where relationships are strongest, we aim to inform targeted resource allocation and cross-sector policy strategies that link public health planning with social welfare and climate adaptation efforts.

## Methods and materials

### Data and variables

#### Dependent measures

We use the prevalence of CHD among adults aged 18 years and older at the census tract level in Los Angeles County as the dependent variable from PLACES: local Data for Better Health 2021 (41). Prevalence of CHD refers to the estimated proportion of adults who have ever been told by a healthcare professional that they had angina or coronary heart disease. PLACES provides model-based small-area estimates at the census-tract level, which we use because direct tract-level measurements of CHD prevalence are not consistently available at comparable spatial resolution across the county. This estimate is derived using a multi-level regression and post-stratification approach applied to Behavioral Risk Factor Surveillance System (BRFSS) and American Community Survey (ACS) data, and calculated by applying predicted probabilities to population estimates at the appropriate geographic level. Although BRFSS is designed to support direct state-level estimation, PLACES leverages BRFSS through this modeling framework to produce tract-level small-area estimates.

#### Key independent measures

##### Social deprivation index

We retrieved the Social Deprivation Index (SDI) from the Robert Graham Center to quantify social deprivation at the census tract level (42–44). SDI is an area-level deprivation measure derived from seven demographic factors from the 2015–2019 ACS 5-year estimate. It serves as a neighborhood-level composite of socioeconomic and housing characteristics, rather than a direct measure of neighborhood amenities or healthcare supply/access. The SDI is calculated using the following seven demographic characteristics collected in the ACS: the percentage of the population (1) living in poverty, (2) with less than 12 years of education, (3) that are single-parent households, (4) living in rented housing units, and (5) living in overcrowded housing (defined as more than one person per room). In addition, the SDI also provided the percentage of (6) households without a car, and (7) non-employed adults under 65 years of age. Together, these factors reflect constrained resources and living conditions that can limit residents' access to health-promoting opportunities. The Robert Graham Center converted ACS indicators to a common centile scale and combined them into a single deprivation factor using factor analysis. The final SDI score is a weighted composite based on the factor loadings of the seven retained indicators (42).

##### Land surface temperature (LST)

We utilized remotely sensed LST data from ECOsystem Spaceborne Thermal Radiometer Experiment on Space Station (ECOSTRESS) because of its high spatial resolution of 70 x 70 meters, frequent capture intervals of 1–5 days, and variable capture times throughout the day. (45) LST is distinct from near-surface air temperature and reflects surface heating and burn potential driven by land cover, imperviousness, and vegetation. Thus, in this study, we interpret ECOSTRESS LST as a surface heat hazard metric rather than as cumulative individual-level heat exposure. To highlight the disparities under extreme heat conditions, we show clear-sky data from a midday overpass (12:44:26 PST) during a heatwave that started on July 12, 2021. The ECOSTRESS data were obtained through the USGS LP-DAAC AppEARS tool. Each LST value, captured in two readings less than a minute apart, was spatially linked to its corresponding area in Los Angeles County, including specific recreational sites, material types, and socioeconomic data at the most granular level available.

### Control variables

We included several sociodemographic control variables at the census tract level from the ACS 5-year estimate (2017–2021): the percentage of the population that is Latiné, Black, Asian, Native Hawaiian, Pacific Islander, foreign-born, over 65 years of age, and has a high school diploma or higher. Census tract-level median household income was also included.

## Statistical analyses

**Figure 1:**
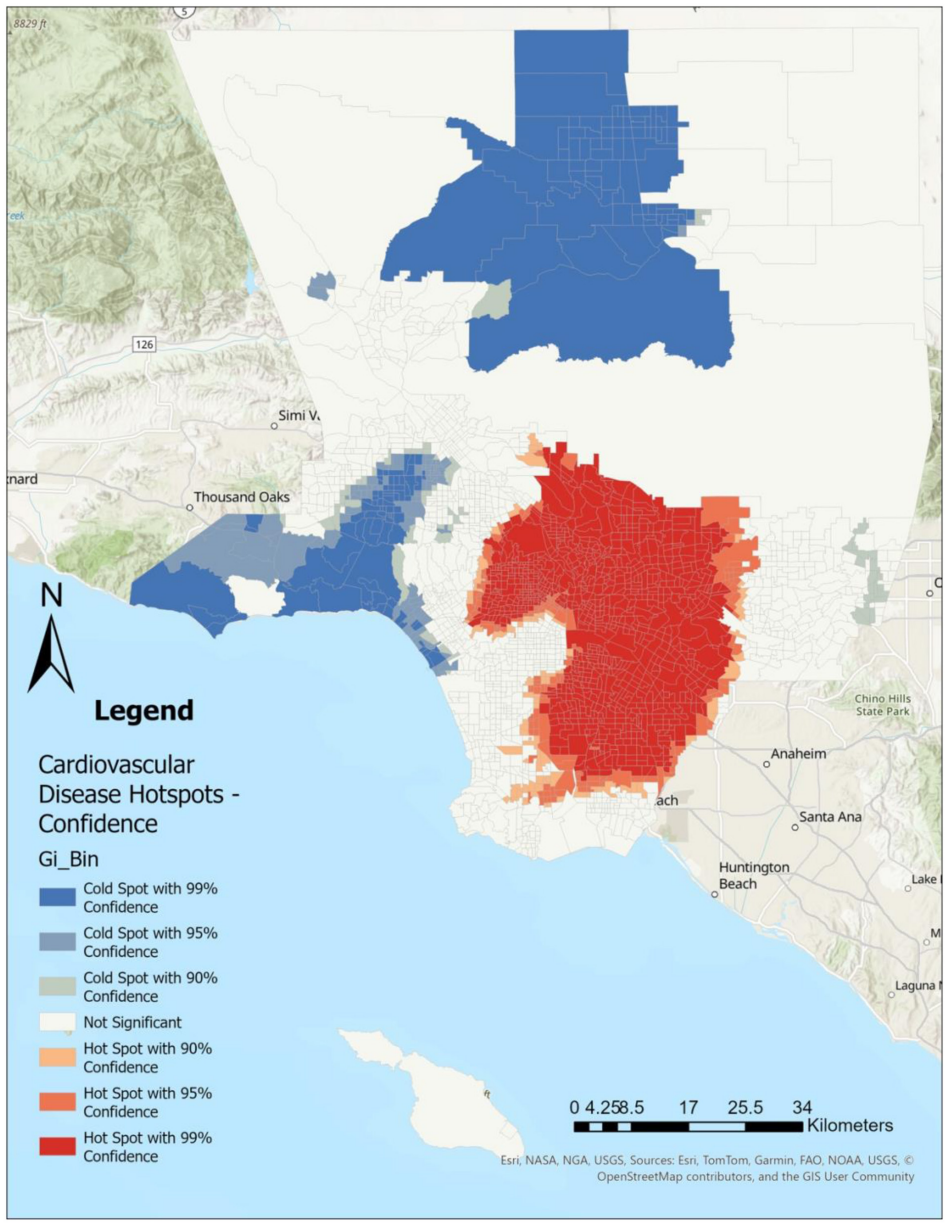
Cardiovascular disease cases are concentrated within southern and eastern portions, whereas low levels of cardiovascular disease can be found in northern and western portions of Los Angeles County. Hot spot analysis using Getis Ord Gi^*^ statistic for cardiovascular disease rates by Los Angeles County census tract, 2021.

**Figure 2:**
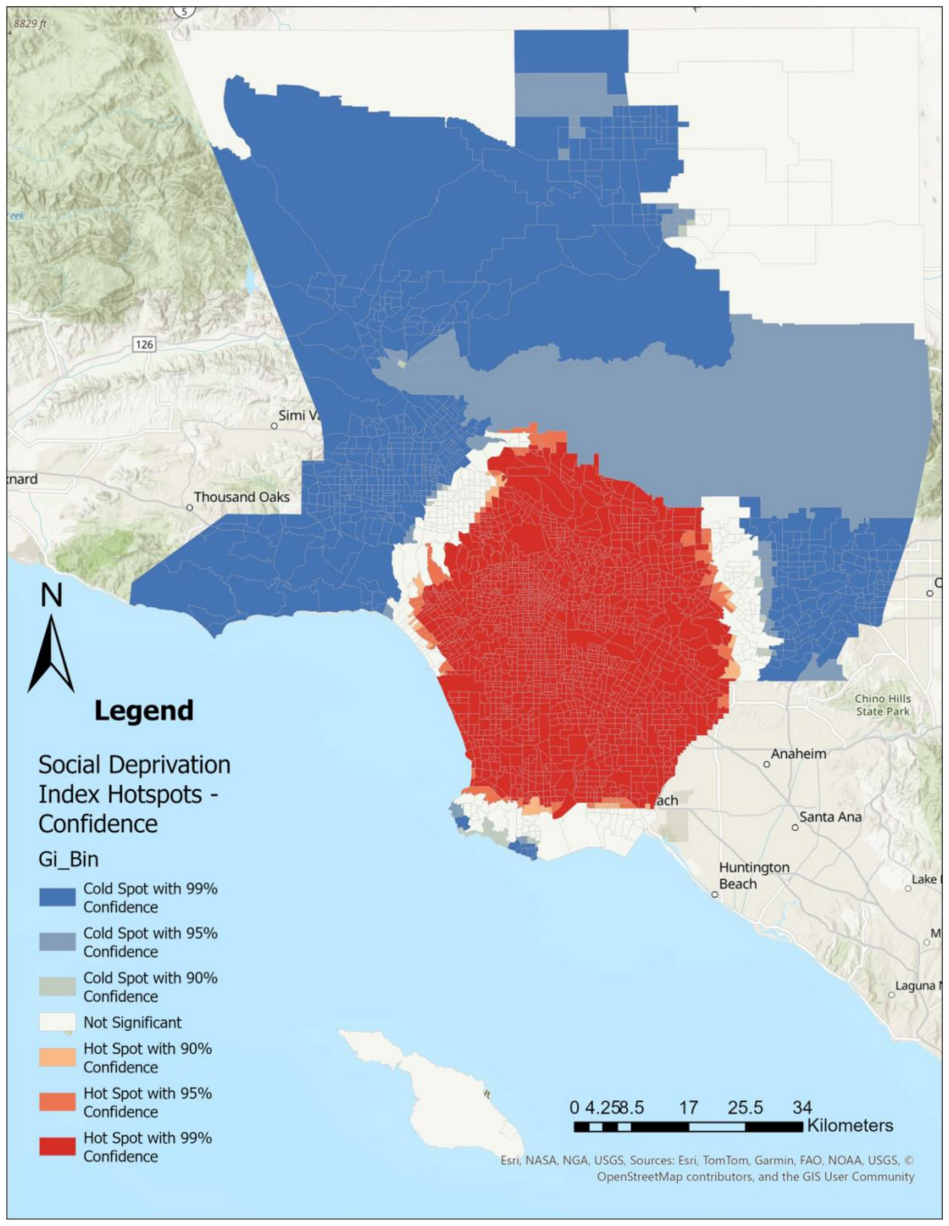
High social deprivation index scores are concentrated throughout southern and eastern Los Angeles County and containing downtown Los Angeles city, while low social deprivation index scores can be found in northern and western parts of the county. Hot spot analysis using Getis Ord Gi^*^ statistic for Social Deprivation Index rates by Los Angeles County census tract, 2021.

**Figure 3:**
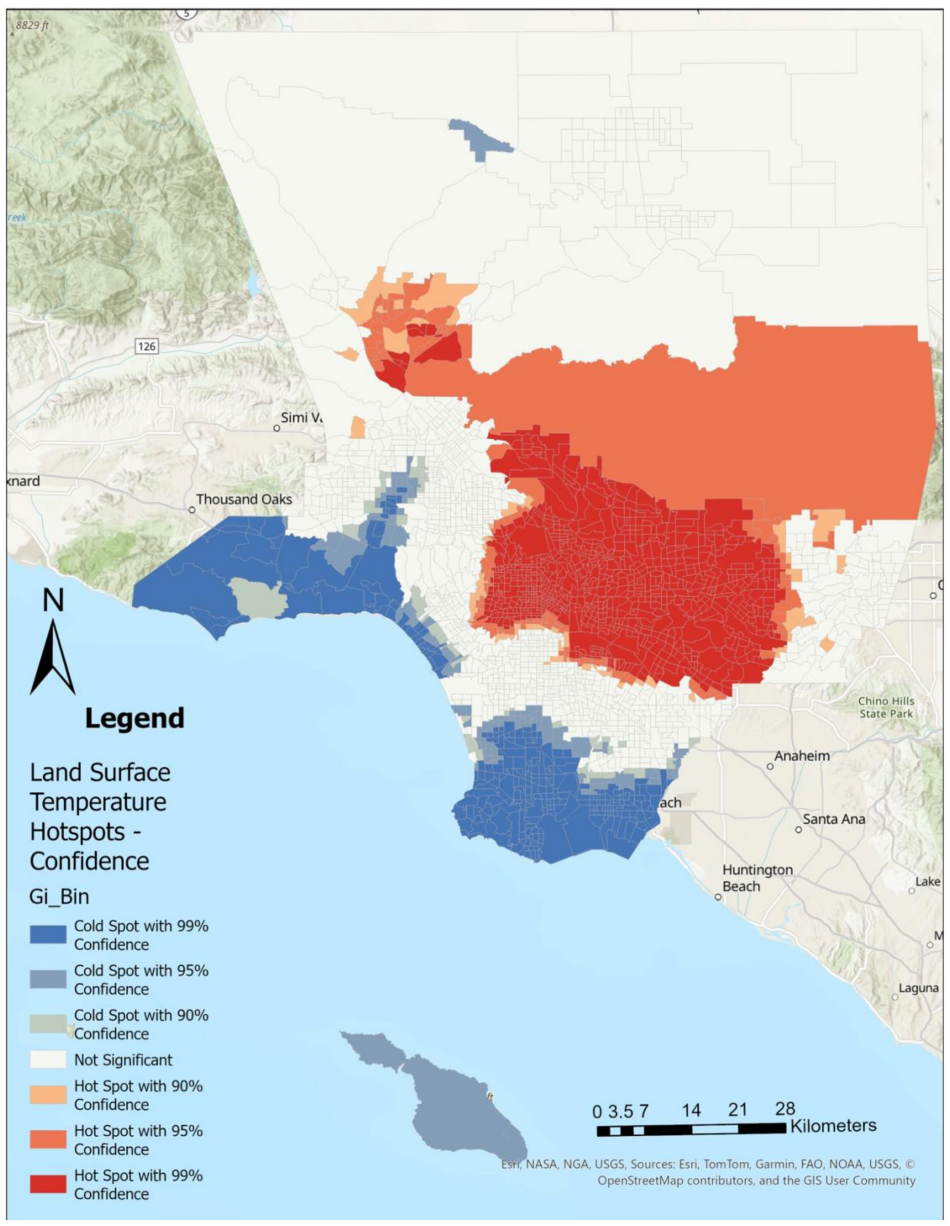
High land surface temperatures are concentrated in eastern and northern parts of the county. Hot spot analysis using Getis Ord Gi^*^ statistic for land surface temperature (Celcius) by Los Angeles County census tract, 2021.

**Figure 4:**
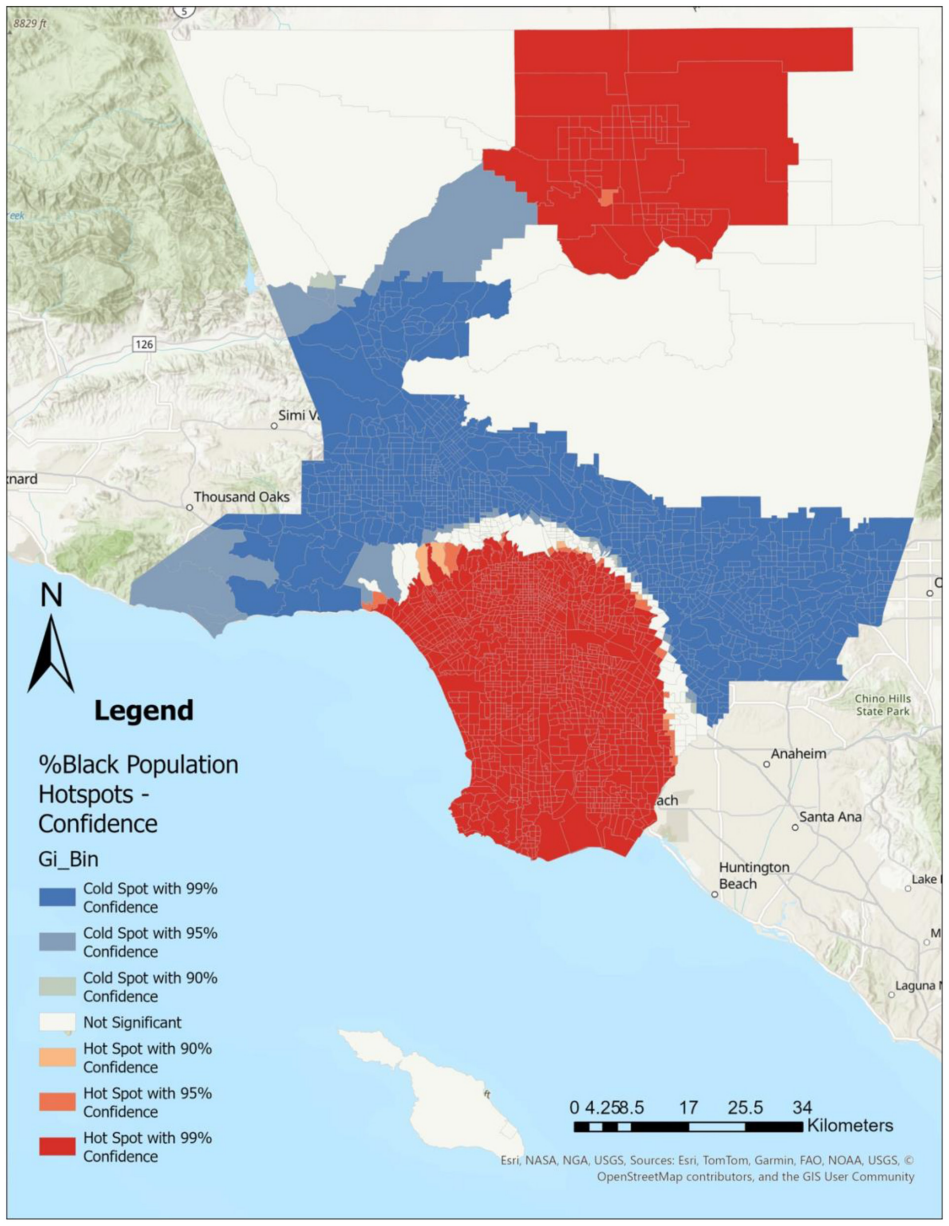
Those identifying as black primarily live in southern and northern parts of Los Angeles County, where in contrast, areas with smaller black populations are primarily in western and far eastern parts of the county. Hot spot analysis using Getis Ord Gi^*^ statistic for the percent Black population by Los Angeles County census tract, 2021.

**Figure 5:**
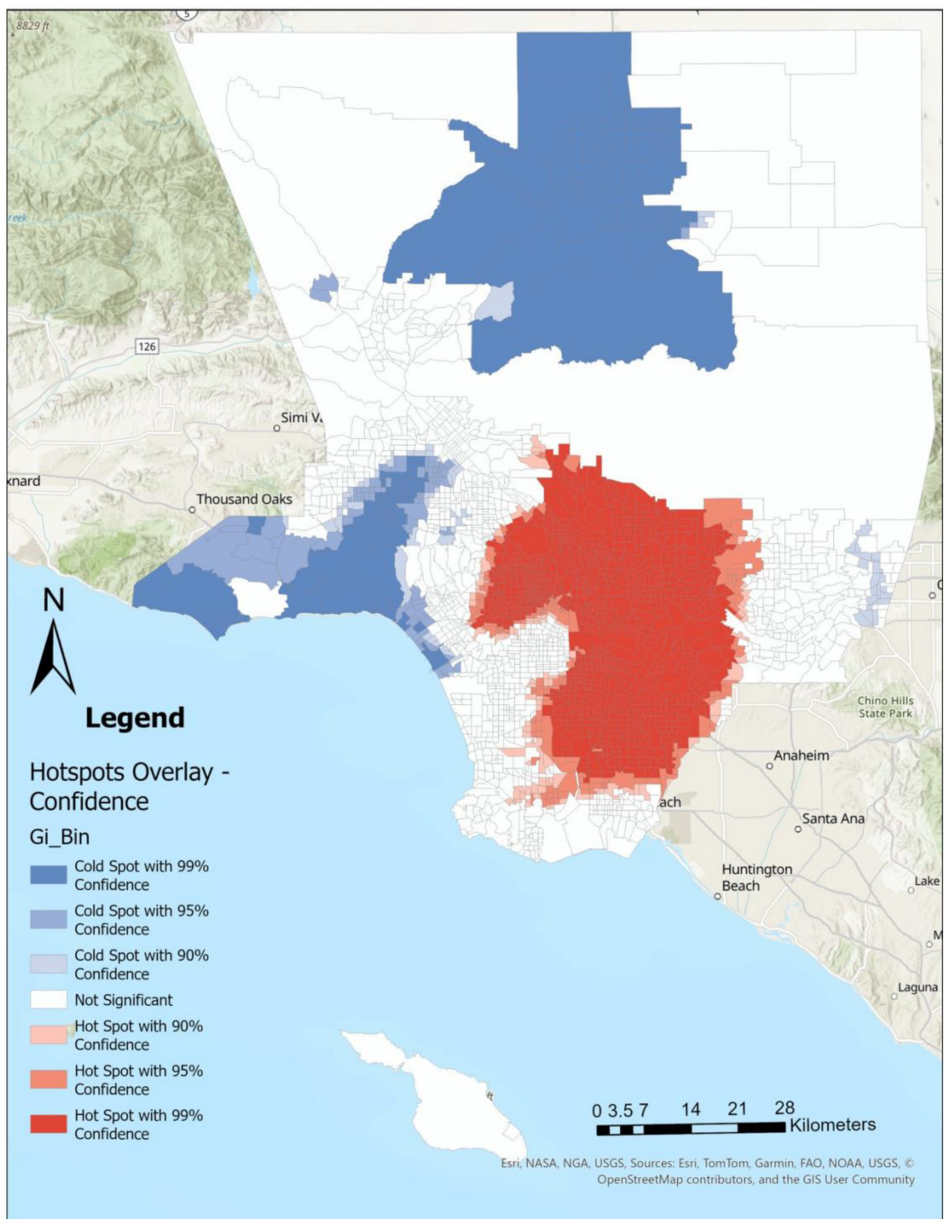
Within southern and parts of eastern Los Angeles County, there are high cardiovascular disease rates, high levels of deprivation, high levels of land surface temperature, and larger percentages of the Black population. Hot spot analysis overlay of cardiovascular disease rate, Social Deprivation Index, Land Surface Temperature, and the percent Black population, all at the census tract level within Los Angeles County to identify common hot and cold spots for each of the variables.

Using Getis-Ord Gi^*^ Optimized Hot Spot Analysis in ArcGIS Pro (*ver 3.4.0*), we identified statistically significant clustering of CHD (Figure 1), SDI (Figure 2), LST (Figure 3), and the percent Black population (Figure 4) within the 2,513 census tracts of Los Angeles County. We created a geospatial overlay (Figure 5) for each variable to pinpoint the intersections of hot spots across all variables. We conducted a hot spot analysis focusing on the percent Black population because it serves as a tract-level indicator of structural racial inequity in the Los Angeles context, where historical segregation and disinvestment have shaped the spatial distribution of environmental burdens and health risks (40).

Next, we developed an ordinary least squares (OLS) regression model with the prevalence of coronary heart disease as the dependent variable and SDI and LST as the main independent variables. We z-score standardized the dependent variable (CHD prevalence) and all numeric independent variables to obtain standardized coefficients that are comparable across predictors. Multicollinearity was assessed using variance inflation factors (VIFs). All predictors showed VIFs below five, indicating no severe multicollinearity (Table 1). The model also controlled for all relevant variables.

**Table 1:** Variance inflation factors (VIFs) for the standardized OLS model.

**Variable**	**VIF**
Socioeconomic deprivation index	2.64
Land surface temperature	1.17
% Black population	1.51
% Hispanic/latino population	4.69
% With high school or higher	4.30
Median household income	2.96
% Foreign born	2.39
% Asian population	2.09
% Aged 65 +	1.43
% Native Hawaiian/Pacific Islander	1.03

We assessed spatial autocorrelation in regression residuals using Moran's I with the same spatial weights matrix. Residuals showed significant positive spatial autocorrelation (Moran's I = 0.0595; z = 4.52; *p* = 3.05 × 10^−6^), indicating remaining spatial dependence. Significant residual spatial autocorrelation indicates that unexplained variation remains spatially clustered and supports the use of spatial models to better account for spatial dependence and potential geographic heterogeneity in associations. Then, we utilized Geographically Weighted Regression (GWR) to account for spatial dependence between SDI, LST, and the prevalence of CHD, as in our prior research (46). This method allowed us to evaluate whether the strength of the association between LST and the prevalence of CHD, as well as with SDI, varied across different locations within Los Angeles County. We used the adaptive bandwidth selection approach, which dynamically adjusts the bandwidth based on the density of data points, ensuring a more accurate representation of spatial variability in urban and rural areas. All GWR analyses were performed in *R*.

## Results

Figures 1–4 illustrate the hot and cold spots of CHD, SDI, LST, and the percentage of the Black population in Los Angeles County census tracts. CHD prevalence exhibits clear geographic clustering, with higher estimated rates concentrated in southern and parts of eastern Los Angeles County. These areas also tend to exhibit higher socioeconomic deprivation and greater surface heat hazard (LST) than other parts of the county. Figure 5 presents an overlay of all the listed variables to identify overlapping hot and cold spots. Notably, within southern and parts of eastern Los Angeles County, CHD hot spots overlap with neighborhoods that have larger percentages of the Black population, highlighting the spatial co-location of cardiovascular burden with structural and environmental disadvantage. We provide descriptive tract characteristics in Table 2.

**Table 2:** LA county census tract (*N* = 2513) characteristics.

**Variables**	**Median**	**Mean**	**SD**	**Min**	**Max**
**Dependent measures**					
Prevalence of coronary heart disease	4.30	4.39	0.88	0.50	9.90
**Key independent measures**					
SDI	71.00	64.59	28.42	2	100
LST	17.97	17.73	1.51	14.51	25.33
**Control variables**					
% Latinx	51.90	50.75	28.21	0	100
% Black	3.23	7.45	11.98	0	86.79
% Asian	9.60	14.57	15.84	0	85.76
% Native Hawaiian and Pacific Islander	0.00	0.01	0.01	0	0.10
% Foreign born	34.66	34.13	12.64	4.61	75.19
% high school or higher education	56.03	55.09	15.07	9.59	91.63
Median household income ($ 1,000 s)	74,844	81,668.29	35,619.33	17,326	250,001
% Ages 65 +	0.13	0.14	0.06	0	0.49

**Figure 6:**
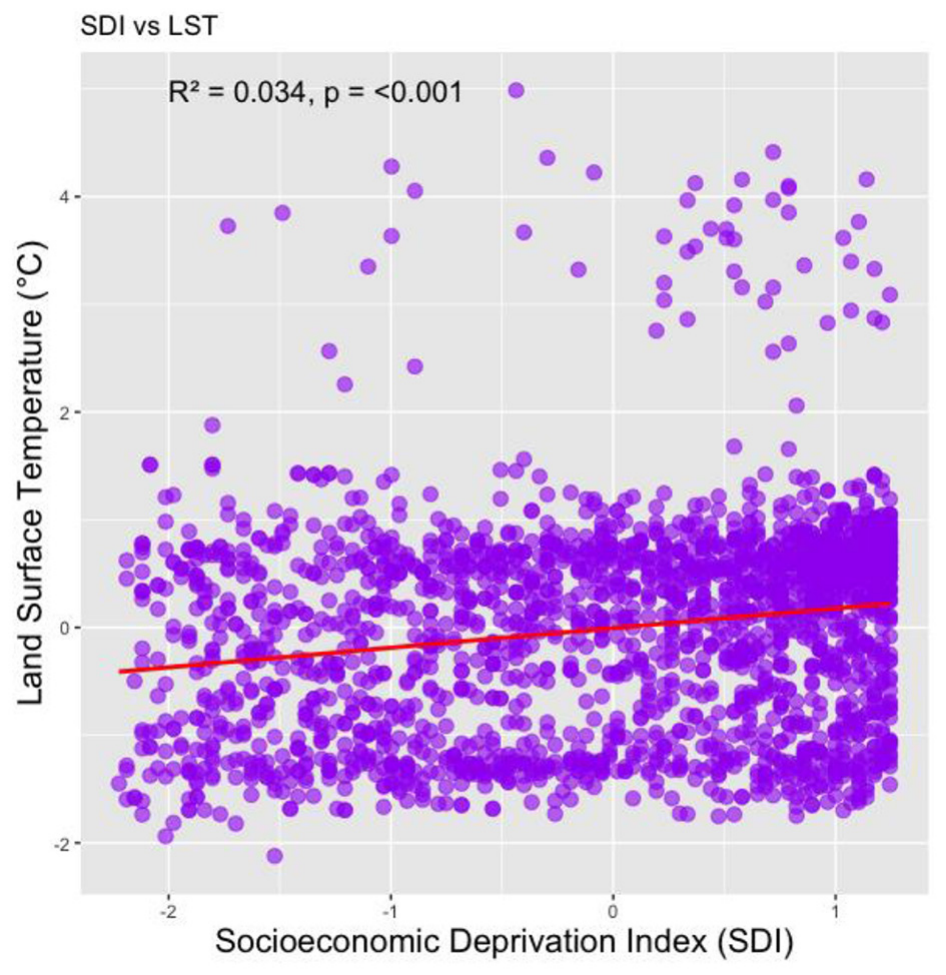
Correlation between Social Deprivation Index (SDI) and Land Surface Temperature (LST). Each point corresponds to a census tract, with LST values plotted against varying levels of SDI. The red regression lines are included to show the general trend.

**Figure 7:**
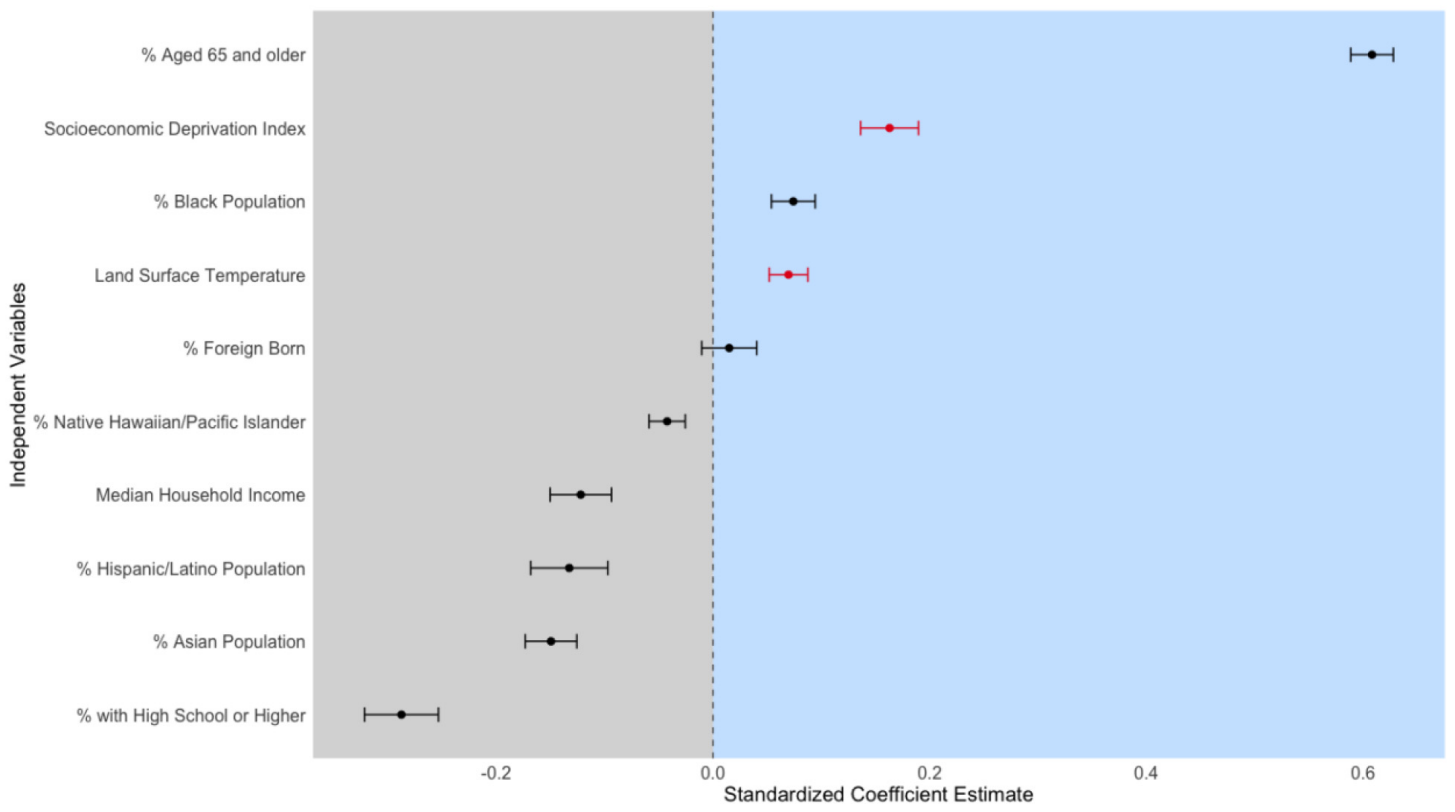
Standardized Coefficient Estimates of the Association Between Socioeconomic and Environmental Factors with Coronary Heart Disease Prevalence in Los Angeles County, Including 95% Confidence Intervals. This figure presents the results of an Ordinary Least Squares (OLS) regression model examining the relationship between LST, SDI, and various socioeconomic and environmental factors and the prevalence of coronary heart disease (CHD) in Los Angeles County. The variables were standardized to allow for comparability across coefficients. The y-axis lists the independent variables, and the x-axis shows the standardized coefficient estimates. Each black dot represents the estimate, with horizontal lines indicating 95% confidence intervals. The shaded areas highlight the regions where coefficient estimates are either negative (left, in gray) or positive (right, in light blue). A vertical dashed line at zero indicates the threshold between negative and positive associations. The key variables, “Socioeconomic Deprivation Index” and “Land Surface Temperature,” are highlighted in red to emphasize their statistically significant positive associations with CHD prevalence.

Figure 6 illustrates the relationship between the SDI and LST in Los Angeles County census tracts. The figure indicates that areas with higher levels of socioeconomic deprivation tend to experience elevated land surface temperatures. Figure 7, Table 3 presents the results of the OLS regression analysis. We found that age over 65, SDI score, percentage of the Black population, and LST have high standardized coefficients. A 1-standard deviation (SD) increase in the SDI is associated with a 0.163-SD higher CHD prevalence, which is statistically significant (*p* < 0.001). Furthermore, a 1 SD Celsius degree increase in heatwave daytime LST is associated with a 0.070-SD higher CHD prevalence (*p* < 0.001).

**Table 3:** Ordinary least squares regression results for prevalence of coronary heart disease in Los Angeles County.

**Variable**	**Estimate**	**Std. error**	***t-*value**
SDI	0.163	0.027	6.093 ^***^
LST	0.07	0.018	3.916 ^***^
% Latinx	−0.133	0.036	−3.725 ^***^
% Black	0.074	0.02	3.669 ^***^
% Asian	−0.15	0.024	−6.282 ^***^
% Native Hawaiian and Pacific Islander	−0.042	0.017	−2.538 ^*^
% Foreign born	0.015	0.025	0.589
% high school or higher education	−0.288	0.034	−8.429 ^***^
Median household income ($ 1,000 s)	−0.122	0.028	−4.314 ^***^
% Ages 65 +	0.608	0.02	30.943 ^***^
(Intercept)	0	0.016	0

^*^ *p* < 0.05; ^***^ *p* < 0.001.

Regarding demographic variables, each SD increase in the percentage of Black residents is associated with 0.074-SD higher CHD prevalence (*p* < 0.001). Conversely, a 1-SD increase in the percentage of Hispanic/Latino residents is associated with a 0.133-SD lower CHD prevalence (*p* < 0.001). The percentage of Asian and Native Hawaiian/Pacific Islander residents is also negatively associated with the health outcome (*p* < 0.05). Educational attainment also demonstrates a significant negative association: a 1-SD increase in the percentage of individuals with at least a high school education in census tracts is associated with a 0.288-SD lower CHD prevalence (*p* < 0.001). Additionally, a 1-SD increase in median household income corresponds to a 0.122-SD lower CHD prevalence (*p* < 0.001). Finally, a 1-SD increase in elderly residents (ages 65+) in the census tracts is associated with a substantial 0.608-SD higher CHD prevalence (*p* < 0.001).

**Figure 8:**
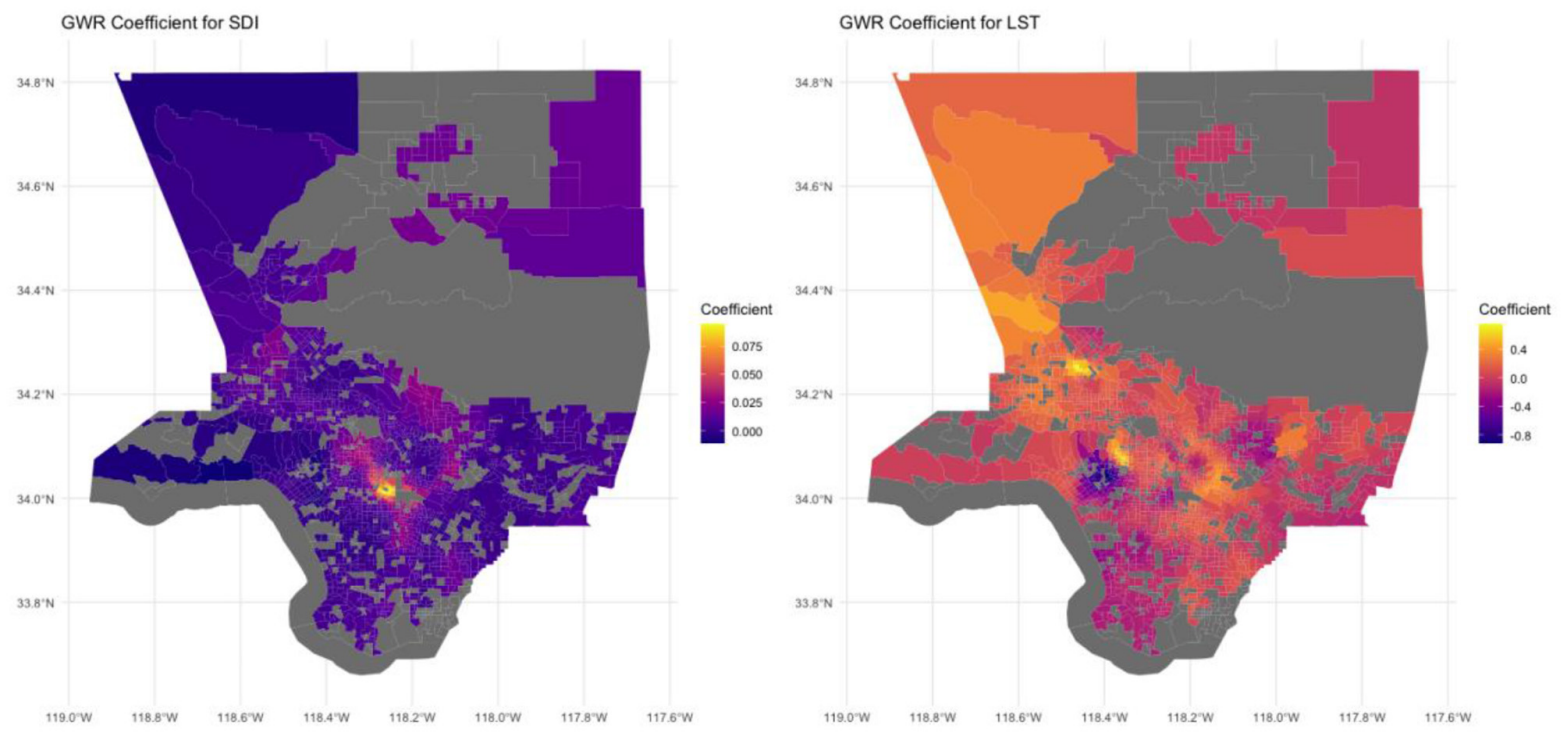
Spatial Distribution of GWR Coefficients for Social Deprivation Index and Land Surface Temperature on Coronary Heart Disease Prevalence in Los Angeles County.

We also applied GWR to examine spatial dependence within the model (Table 4). The GWR model accounted for a significant portion of the variance (*R*^2^ = 0.74), indicating that accounting for spatial heterogeneity enhanced the model's explanatory power. The AIC is 3,410, which is lower than that of the global regression (AIC = 5,392), suggesting that the GWR model offers more explanatory strength than the OLS model by factoring in spatial variation. Figure 8 displays the GWR coefficients for the SDI and LST across census tracts in Los Angeles County. The left panel illustrates the GWR coefficients for SDI, where regions with higher coefficients (yellow) indicate a stronger positive relationship between social deprivation and CHD prevalence. These high SDI coefficients are primarily concentrated in central and southern Los Angeles, implying that increased social deprivation is closely correlated with higher CHD prevalence in these areas. In contrast, northern and western areas, depicted by darker purple, show weaker or no associations between SDI and CHD, suggesting that socioeconomic deprivation plays a lesser role in influencing health outcomes in these regions. The right panel presents the GWR coefficients for LST, with warmer colors (orange and red) signifying a stronger positive association between higher temperatures and CHD prevalence. The central and eastern regions of Los Angeles County exhibit exceptionally high coefficients, reflecting a notable increase in CHD prevalence with rising heatwave daytime LST in these areas. Cooler regions (purple), predominantly in the southern parts of the county, demonstrate negative or weak associations between LST and CHD.

**Table 4:** Geographically weighted regression (GWR).

**Variable**	**Min**	**1st Qu**.	**Median**	**3rd Qu**.	**Max**
SDI	−0.364	0.043	0.159	0.358	3.136
LST	−1.569	−0.125	0.042	0.192	1.851
% Latinx	−2.061	−0.503	−0.261	0.287	2.615
% Black	−2.806	−0.558	−0.113	0.138	1.781
% Asian	−1.397	−0.529	−0.277	0.063	1.96
% Native Hawaiian and Pacific Islander	−0.483	−0.071	−0.026	0.031	0.624
% Foreign born	−1.062	−0.214	−0.055	0.161	1.096
% high school or higher education	−1.516	−0.46	−0.207	0.075	1.123
Median household income ($ 1,000 s)	−1.674	−0.446	−0.121	0.057	0.474
% Ages 65 +	−0.008	0.247	0.498	0.802	1.595
(Intercept)	−3.445	−0.636	−0.267	−0.03	1.629

Results: Spatial variability of factors associated with coronary heart disease prevalence.

## Discussion

This study enhances our understanding of environmental health disparities by illustrating the relationship between socioeconomic deprivation, LST, and prevalence of CHD in Los Angeles County. Our analysis revealed that census tracts with higher levels of social deprivation and increased LST were linked to a greater prevalence of coronary heart disease (CHD). Significant associations between social deprivation, LST, and CHD prevalence persisted even after accounting for demographic and socioeconomic factors. These findings indicate that environmental (LST) and socioeconomic factors are critical yet modifiable determinants of health, exhibiting spatial variability across the county. We discuss the implications of these results for public health interventions and policies aimed at reducing health disparities in socially deprived and environmentally vulnerable communities.

Our findings confirm that socioeconomic deprivation, as measured by the SDI, and environmental factors, such as LST, are significantly associated with the prevalence of CHD. These results align with the broader literature on social determinants of health, which consistently shows that neighborhoods with higher levels of deprivation face greater burdens of chronic diseases due to factors like reduced access to healthcare, unhealthy lifestyle behaviors, and environmental stressors (47). The observed relationship between LST and CHD prevalence adds to the growing evidence that environmental heat exposure, particularly in socially disadvantaged urban areas, exacerbates health risks, especially cardiovascular conditions (30). This aligns with the “urban heat island” effect, which disproportionately impacts low-income and minority communities (48). The demographic disparities observed, such as the higher CHD prevalence in majority Black neighborhoods, further illustrate the intersection of racial, socioeconomic, and environmental inequalities in shaping health outcomes. These findings underscore the urgent need for targeted, community-engaged public health interventions that address structural and environmental health determinants, focusing on improving conditions in vulnerable communities (49).

The findings from this study have significant public health policy implications and highlight the urgent need for collaboration with community partners to ensure effective interventions. First, they emphasize the necessity for targeted interventions in neighborhoods facing high levels of social deprivation and environmental stressors, such as elevated LST, which disproportionately affect low-income communities and communities of color, particularly Black and Latiné populations (48, 50). Urban planning initiatives—like increasing green spaces, improving housing conditions, and expanding access to healthcare—must prioritize these areas. Evidence suggests that urban greening is associated with a reduced risk of cardiovascular disease through stress reduction, lower air pollution, and increased physical activity, while housing improvements, including insulation and ventilation upgrades, have been linked to better cardiovascular outcomes and a reduction in health disparities (51, 52). The interventions should also actively involve the local organizations and residents to ensure these changes meet community needs (46, 49). Incorporating residents and community organizations through community-based participatory research ensures that interventions align with community priorities and enhance participation (53). This approach also builds local capacity and supports structural changes that advance health equity (54). Second, the notable spatial variability identified in the GWR model indicates that a one-size-fits-all approach is unlikely to adequately address health disparities. Instead, collaborating closely with community partners to develop localized strategies that account for the distinct social and environmental contexts of different neighborhoods will be vital in reducing the burden of cardiovascular disease.

Our study is not without limitations. The observational nature of the study design limits the power to estimate causal inferences, as unmeasured confounding factors may influence the observed relationships. Our analysis is also geographically confined to LA County. While this allows for a focused examination of the region, it may restrict the generalizability of our findings to other areas with different social and environmental contexts. Also, because PLACES outcomes are modeled as small-area estimates, the underlying smoothing/modeling may dampen local extremes and influence spatial clustering and residual dependence. Furthermore, because SDI is constructed from aggregated socioeconomic and housing indicators, it captures upstream deprivation rather than directly measuring the distribution of specific neighborhood amenities or healthcare facilities. Additionally, the ECOSTRESS LST metric represents a single clear-sky midday overpass during an extreme heat event, which captures spatial patterns of peak surface heating but not cumulative heat exposure (55). Finally, the LST data may not fully capture individuals' exposure, particularly in indoor environments and with personal mitigative measures. Therefore, it should be interpreted as a surface heat hazard indicator rather than an individual-level exposure metric.

Thus, in the next step, we plan to utilize a longitudinal study design to monitor health outcomes over time, thereby providing stronger evidence for the causal relationship between social deprivation, LST, and health disparities. We will also construct multi-date warm-season composites and integrate air temperature/heat index products to better represent chronic exposure. Additionally, literature suggests that reducing heat in urban areas through cool pavements and green spaces may help address the issue (56). Future studies should also investigate the effectiveness of specific urban planning and public health interventions aimed at alleviating the negative impacts of high LST in socially deprived communities. Such interventions could encompass the development of green infrastructure and community health initiatives tailored to the unique needs of these neighborhoods.

## Conclusion

This study demonstrates that socioeconomic deprivation and elevated heat are significant and spatially variable predictors of cardiovascular disease prevalence in Los Angeles County. By integrating high-resolution environmental data with tract-level social and demographic indicators, we found that both SDI and heatwave daytime LST contribute independently and synergistically to cardiovascular health disparities, particularly in communities of color and areas with lower income and educational attainment. Notably, the effects of these factors are not uniform across the county, with central and southern regions showing stronger associations, underscoring the importance of spatially targeted interventions. These findings highlight the urgent need for equity-driven urban planning and public health strategies that simultaneously address the structural roots of deprivation and the unequal distribution of environmental burdens. Mitigating cardiovascular health disparities in urban settings will require coordinated action across public health, climate resilience, and community development sectors.

## Data Availability

The original contributions presented in the study are included in the article/supplementary material, further inquiries can be directed to the corresponding author. ECOSTRESS data are available from: https://appeears.earthdatacloud.nasa.gov/.
